# Human spinal cord tissue is an underutilised resource in degenerative cervical myelopathy: findings from a systematic review of human autopsies

**DOI:** 10.1007/s00701-023-05526-5

**Published:** 2023-02-23

**Authors:** Esmee Dohle, Sophie Beardall, Aina Chang, Karla P. Corral Mena, Luka Jovanović, Upamanyu Nath, Keng Siang Lee, Alexandria H. Smith, Arun J. Thirunavukarasu, Alvaro Yanez Touzet, Emma Jane Norton, Oliver D. Mowforth, Mark R. N. Kotter, Benjamin M. Davies

**Affiliations:** 1 School of Clinical Medicine, University of Cambridge, Cambridge, UK; 2 School of Medical Sciences, Faculty of Biology, Medicine and Health, The University of Manchester, Manchester, UK; 3 North Manchester General Hospital, Manchester University NHS Foundation Trust, Manchester, UK; 4 Department of Basic and Clinical Neurosciences, Maurice Wohl Clinical Neuroscience Institute, Institute of Psychiatry, Psychology and Neuroscience (IoPPN), King’s College London, London, UK; 5 Department of Neurosurgery, King’s College Hospital, London, UK; 6 Division of Anaesthesia, Addenbrooke’s Hospital, University of Cambridge, Cambridge, CB2 0QH UK; 7 Department of Clinical Neurosciences, University of Cambridge, Cambridge, UK

**Keywords:** Degenerative cervical myelopathy, Ossification posterior longitudinal ligament, Autopsy, Histology, Pathophysiology, Systematic review

## Abstract

**Study design:**

Systematic review.

**Background:**

Although degenerative cervical myelopathy (DCM) is the most prevalent spinal cord condition worldwide, the pathophysiology remains poorly understood. Our objective was to evaluate existing histological findings of DCM on cadaveric human spinal cord tissue and explore their consistency with animal models.

**Methods:**

MEDLINE and Embase were systematically searched (CRD42021281462) for primary research reporting on histological findings of DCM in human cadaveric spinal cord tissue. Data was extracted using a piloted proforma. Risk of bias was assessed using Joanna Briggs Institute critical appraisal tools. Findings were compared to a systematic review of animal models (Ahkter et al. 2020 Front Neurosci 14).

**Results:**

The search yielded 4127 unique records. After abstract and full-text screening, 19 were included in the final analysis, reporting on 150 autopsies (71% male) with an average age at death of 67.3 years. All findings were based on haematoxylin and eosin (H&E) staining. The most commonly reported grey matter findings included neuronal loss and cavity formation. The most commonly reported white matter finding was demyelination. Axon loss, gliosis, necrosis and Schwann cell proliferation were also reported. Findings were consistent amongst cervical spondylotic myelopathy and ossification of the posterior longitudinal ligament. Cavitation was notably more prevalent in human autopsies compared to animal models.

**Conclusion:**

Few human spinal cord tissue studies have been performed. Neuronal loss, demyelination and cavitation were common findings. Investigating the biological basis of DCM is a critical research priority. Human spinal cord specimen may be an underutilised but complimentary approach.

**Supplementary information:**

The online version contains supplementary material available at 10.1007/s00701-023-05526-5.

## Introduction

Degenerative cervical myelopathy (DCM) is a disabling neurological condition in which degenerative changes to the cervical spine stress and injure the spinal cord [16]. It is considered the most common spinal cord condition worldwide [13], with a recent meta-analysis of imaging studies estimating a prevalence of 2.3% [49]. DCM is also known around the world by many different names. The term DCM was proposed to unify terminology [40], as an umbrella term for subtypes of pathology such as cervical spondylotic myelopathy (CSM) and ossification of the posterior longitudinal ligament (OPLL), and replacement for synonymous terms such as cervical stenosis or disc herniation with myelopathy. This has recently been endorsed in a global consensus process called AO Spine RECODE-DCM [15].

Currently, the pathophysiology of DCM is poorly understood [16]. In DCM, chronic compression of the spinal cord by degenerative and aberrant structures leads to both white and grey matter damage. This leads to progressive neurological dysfunction, such as sensory deficits including hypoaesthesia, paraesthesia and allodynia, loss of dexterity, incontinence and tetraplegia [13]. However, the precise mechanisms through which compression causes this damage are unclear. Furthermore, although approximately 1 in 5 adults have asymptomatic spinal cord compression on MRI, only a proportion with spinal cord compression progress to DCM, indicating that in most cases, spinal cord compression does not cause DCM [49].

Most mechanistic insights have arisen from a small number of pre-clinical studies, including goat [26], rabbit [61], rodent [35, 58] or canine models [5]. Generally, injury has been replicated by insertion of screws, balloons or expandable polymers. One exception is the *Twy twy* mouse model, in which hyperosteosis causes a high cervical stenosis. In these studies, macroscopic findings include venous congestion, ischaemia and oedema [5, 35]. Cellular changes demonstrated in animal models include loss of motor- and interneurons, axon degeneration, gliosis and demyelination [26, 61, 58, 31, 28, 59, 3]. However overall, it has been difficult to simulate a truly chronic injury, with the diverse range of degenerative features (e.g. anterior and posterior compression) seen in DCM.

Human autopsy therefore presents an important alternative. While a series by Ito et al. is well cited [24], the clinical literature on DCM human tissue has not been systematically searched or aggregated and it is uncertain whether other sources exist. Therefore, the objective of this study was to systematically identify studies with histological findings of DCM from human spinal cord specimens, and to aggregate their findings. We also aimed to compare their findings to existing pre-clinical studies.

## Methods

### Search strategy

A search strategy was developed which combined existing search filters for DCM [14], [30] with synonyms for autopsy, cadaver and histopathology, with oversight from a medical librarian. The search was performed using OVID (Wolters Kluwer, Netherlands) from inception to 6 May 2022, and applied to MEDLINE and Embase. The search was prospectively registered with PROSPERO (CRD42021281462, Supplementary Data 1). The search strategy as applied to MEDLINE and Embase can be found in Supplementary Data 2.

### Study selection

The sensitive search strategy yielded 4127 records after removal of duplicates. Titles and abstracts were independently screened by at least two reviewers out of a group of eight (ED, SB, AC, KCM, LJ, UN, AS, AJT) using blinding via Rayyan [42]. This was preceded by a pilot screen of 193 records (5% of total) which were screened by all eight reviewers to ensure concordance and to resolve any potential misunderstandings over inclusion and exclusion criteria. Disagreements were resolved by consensus or discussion with a senior reviewer (BD).

### Inclusion and exclusion criteria

Primary research studies which included findings from the spinal cord specimen of humans with DCM were included. This included cervical spondylotic myelopathy (CSM) and cervical myelopathy secondary to ossification of the posterior longitudinal ligament (OPLL). Articles published in a foreign language or without full text were excluded.

### Data extraction and analysis

Data was extracted from included studies using a piloted proforma which included study details, study type, patient demographics, diagnosis, methods and pathological findings on autopsy. For the purpose of this review, ‘autopsy’ refers to a single human spinal cord examined histologically. As most included studies were case reports or case series, the Joanna Briggs Institute (JBI) critical appraisal tools were used to assess the quality of included studies. For analysis, pathological findings for autopsies in case reports and case series were categorised into demyelination, axon loss, necrosis, cavitation, haemorrhage, gliosis and neuronal loss. These overarching categories were taken from the literature, and chosen to aid comparison with pre-clinical studies [3]. Subsequently, each study was scored according to whether a finding within each subgroup was reported. Graphs were produced in R using the ggplot2 package [55]. Ninety-five percent confidence interval was estimated using binomial calculation. Schematics were created using Inkscape (http://www.inkscape.org/).

### Public involvement

This systematic review aligns with the AO Spine RECODE-DCM, Research Priority number 5, investigating the biological basis of DCM [16]. This priority was established with people living with DCM [11]. The conduct of this individual review did not involve members of the public.

## Results

### Study summary

#### Search results

**Fig. 1:**
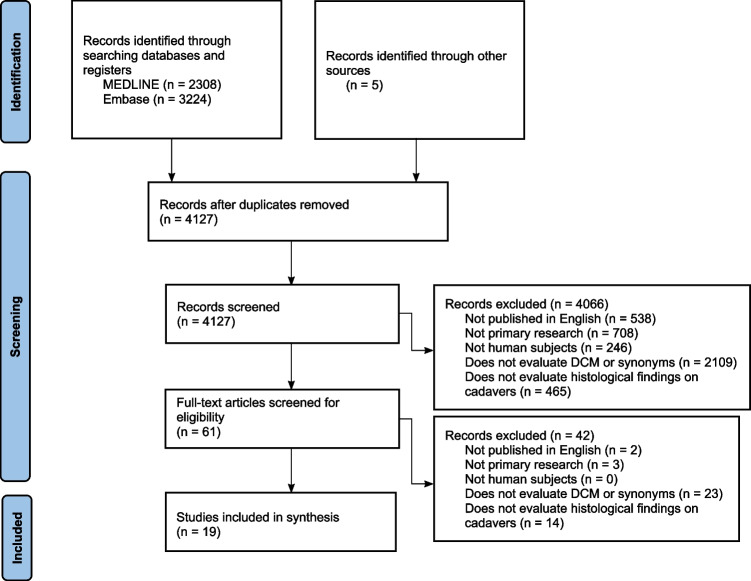
PRISMA diagram

Our search identified a total of 5532 records (2308 in MEDLINE, 3224 in Embase, 5 from other sources), with 4127 remaining after deduplication. A total of 61 articles were selected for full-text screening, of which 19 were included in the final analysis. A full Preferred Reporting Items for Systematic Reviews and Meta-Analyses (PRISMA) flow chart is shown in Fig. 1.

#### Study characteristics and patient demographics

This systematic review included 13 case series and 6 case reports. A total of 150 autopsied patients were included, of whom 71% was male and 29% female and with an average age at death of 67.3. Patient diagnoses within the umbrella of DCM included cervical spondylotic myelopathy (CSM, 13 papers or 68%) and ossification of the posterior longitudinal ligament (OPLL, 6 papers or 32%). An overview of study properties and patient characteristics is shown in Table 1.

**Table 1:** Overview of study characteristics and patient demographics

Property	Number	%
Total studies included	19	100
Case reports	6	32
Case series	13	68
Total patients autopsied	150	100
Male	107	71
Female	43	29
Average age at death	67.3	-
Publication year		
Maximum year of publication	2011	-
Minimum year of publication	1952	-
Country of publication, by number of patients		
Japan	134	89
Canada	8	5.3
UK	7	4.6
USA	1	< 1

### Pathological findings

#### Neuronal loss

**Fig. 2:**
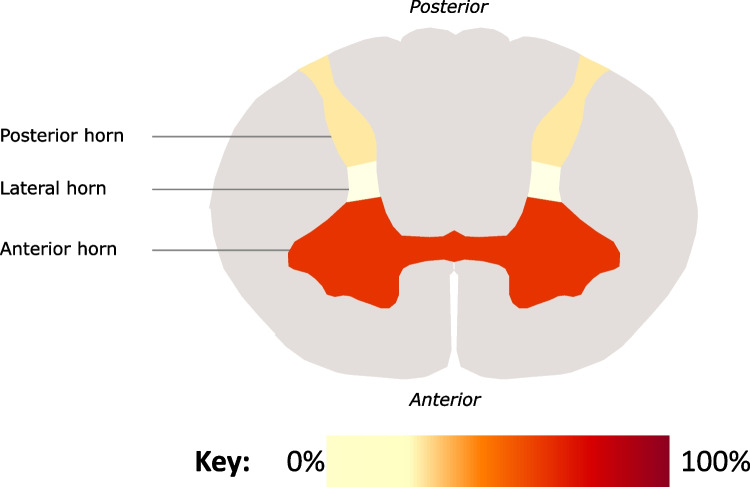
Heat map of common locations of neuronal loss in included studies (*n* = 17)

The most common pathological finding was loss of neuronal cell bodies, with 17 studies and a total of 71 autopsies reporting this (46% of autopsies on CSM and 59% of autopsies on OPLL). The primary location of neuronal loss was variable. A total of 12 studies reported that neuronal loss appeared to primarily affect the anterior horns, whereas 5 studies suggested the posterior horns of which 2 studies also noted lateral horn involvement (Fig. 2). There are some indications that the site of neuronal loss may be associated with disease severity. For instance, one case series which autopsied seven patients with cervical spondylotic myelopathy (CSM) found that the anterior horns were affected in all patients, but only in the most severe cases was neuronal loss also found in the posterior horn [24]. Another study correspondingly indicated that the anterior horns are more immediately vulnerable to dural sac indentations [25]. Only one study, however, compared histological findings of DCM on human spinal cord to healthy controls: this indicated that neuronal loss in the anterior horn is observed in patients with DCM but not in healthy controls [48]. All findings were based on haematoxylin and eosin (H&E) staining, with some studies using the Kluver-Barrera method.

#### Cavitation

Another key finding on autopsy of DCM patients was cavitation, with 14 studies and a total of 33 autopsies reporting this (21% of autopsies on CSM and 29% of autopsies on OPLL). This was most commonly described as cystic and related to areas of degeneration. Several different studies were able to correlate the formation of a cystic cavity with the radiological finding of ‘snake-eyes appearance’ on MRI. [37, 38, 50]. In particular, Mizuno et al. (2003, 2005) reported that this snake-eyes appearance could be a result of cystic necrosis occurring secondary to mechanical compression and venous infarction. Pressure of this cystic cavity on remaining surrounding neurons was associated with destruction of the grey matter. Therefore, the radiological finding of snake-eye appearance is likely to be an unfavourable prognostic factor, as it indicates damage visible histopathologically [37, 38].

#### Demyelination and axon loss

White matter changes were also widespread in the included autopsies. Demyelination was reported in 15 studies and a total of 45 autopsies (27% of autopsies on CSM and 53% of autopsies on OPLL). Correspondingly, axon loss was also reported in 13 included studies and a total of 42 autopsies (25% of autopsies on CSM and 53% of autopsies on OPLL). However, most studies simply reported ‘demyelination’, ‘myelin pallor’ or ‘reduced myelin’, meaning it could not be confidently assessed whether this reflects a process of primary demyelination or a general process of axon loss and degeneration.

The location of white matter changes was variable, with some indicating pathology was most significant in the posterior and lateral funiculus [50], while most studies reported white matter degeneration was present throughout. Descriptions of axon loss were confined to white matter. All findings were based on haematoxylin and eosin (H&E) staining, with some studies using the Kluver-Barrera method with Luxol fast blue staining to visualise myelin.

#### Gliosis

Gliosis was reported in 11 included studies and a total of 39 autopsies (27% of autopsies on CSM and 18% of autopsies on OPLL). All findings were based on haematoxylin and eosin (H&E) staining, with some studies using the Kluver-Barrera method.

#### Necrosis

Necrosis was reported in 7 studies and a total of 19 autopsies (12% of autopsies with CSM and 25% of autopsies with OPLL). The strength of evidence for this was poor: no studies performed quantification, and either haematoxylin and eosin (H&E) and Kluver-Barrera staining methods were used to visualise necrosis across studies. An overview of included studies and their reported findings is shown in Table 2.

**Table 2:** Overview of included studies

Authors	Year	Country	Study type	Sample size	Age (mean)	Diagnosis	Pathological findings
Hawkins et al	1978	USA	Case report	1	48	CSM	Demyelination, axon loss, cavitation, gliosis and neuronal loss
Mizuno et al	1999	Japan	Case report	1	63	OPLL	Demyelination, axon loss, cavitation and neuronal loss
Mizuno et al	2003	Japan	Case series	9	57	CSM	Necrosis, cavitation, gliosis and neuronal loss
Mizuno et al	2005	Japan	Case report	1	73	OPLL	Cavitation and neuronal loss
Ono et al	1977	Japan	Case series	2	58	OPLL	Demyelination
Shiraishi et al	1996	Japan	Case series	7	81	CSM	Demyelination, axon loss, cavitation and neuronal loss
Shimizu et al	2008	Japan	Case series	11	70	CSM	Gliosis and neuronal loss
Someya et al	2011	Japan	Case report	1	65	CSM	Axon loss, cavitation and neuronal loss
Ito et al	1996	Japan	Case series	7	68	CSM	Demyelination, axon loss, cavitation, gliosis and neuronal loss
Iwabuchi et al	2004	Japan	Case series	68	76	CSM	Demyelination, necrosis, cavitation, gliosis and neuronal loss
Payne et al	1957	UK	Case series	2 (DCM patients)	NA	CSM	Demyelination
Ogino et al	1983	Japan	Case series	9	76	CSM	Demyelination, axon loss, necrosis, cavitation and neuronal loss
Hashizume et al	1984	Japan	Case series	3	62	OPLL	Demyelination, axon loss, necrosis, cavitation, gliosis and neuronal loss
Kameyama et al	1995	Japan	Case series	9	64	OPLL	Demyelination, axon loss, necrosis, cavitation and neuronal loss
Bedford et al	1952	UK	Case report	1	71	CSM	Demyelination, axon loss, gliosis and neuronal loss
Mair et al	1953	UK	Case series	4	58	CSM	Demyelination, axon loss, necrosis, cavitation, gliosis and neuronal loss
Ono et al	1977	Japan	Case series	5	74	CSM	Demyelination, axon loss, necrosis, cavitation, gliosis and neuronal loss
Yu et al	2011	Canada	Case series	8	73	CSM	Demyelination, axon loss, cavitation, gliosis, neuronal loss
Murakami et al	1978	Japan	Case report	1	75	OPLL	Demyelination, axon loss, gliosis, neuronal loss

### Comparison between OPLL and CSM

**Fig. 3:**
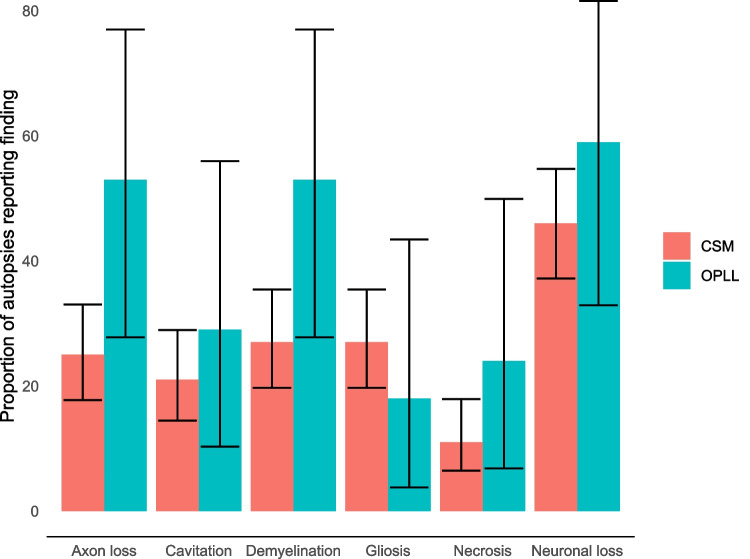
Proportion of autopsies reporting pathological findings. Error bars reflect 95% CI. For CSM, *n* = 133 cases. For OPLL, *n* = 17 cases

The relative prevalence of findings amongst CSM and OPLL autopsies is shown in Fig. 3. While demyelination and axon loss appeared more prevalent in CSM, estimated 95% confidence intervals are overlapping indicating that this is not certainly a significant difference.

### Comparison to pre-clinical models of DCM

**Fig. 4:**
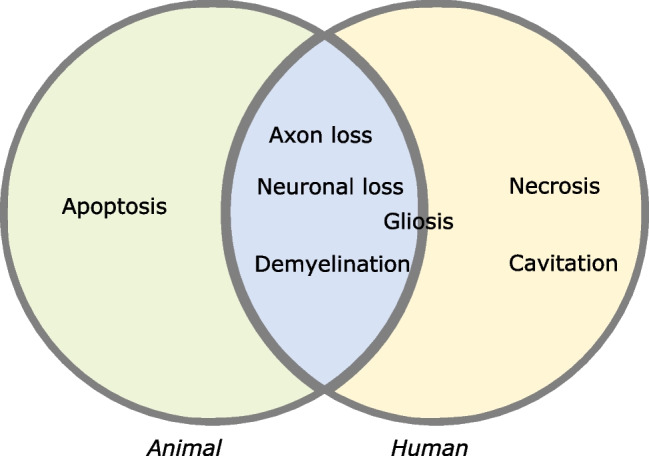
Venn diagram of findings commonly reported in human autopsy and animal model studies of DCM

A systematic review by Akter et al. investigated pathological findings of DCM reported aggregate findings from animal models. When comparing this to our human autopsy findings, it is notable that although animal and human studies both commonly report neuronal loss, demyelination and axon loss, human studies tend to report necrosis whereas animal studies report apoptosis. Additionally, human studies frequently report cavitation. Furthermore, the role of glial cells in animal studies appears more variable, with reports of both glial cell proliferation and glial cell loss. In contrast, human studies commonly report gliosis (Fig. 4).

## Discussion

This systematic review investigated the histological findings of degenerative cervical myelopathy in human spinal cord from autopsy. Few spinal cord specimens have been studied, the vast majority of which used only basic staining techniques such as H&E and Kluver-Barrera. Only one study also used more sophisticated immunohistochemical techniques involving antibodies such as anti-Fas and anti-CD68 [60]. Of the seven predefined histological features of DCM, only haemorrhage was not observed. The most common finding was neuronal loss, but also cavitation, demyelination, axon loss, gliosis and necrosis. Most studies reported the minimum involvement of the anterior horn, with one study linking both anterior and posterior horn involvement to more severe disease. This, alongside the relatively increased reports of cavitation and necrosis, reflects differences when compared to animal models.

### Comparison with animal studies

The significance of the observed differences between animal and human autopsy findings are uncertain.

That many findings were consistent, would certainly support the validity of pre-clinical models. For example, the most common finding in this systematic review of neuronal loss has also been well-documented in animal studies on DCM [7, 22, 29, 31]. Similarly, axonal loss has been demonstrated in small-animal experimental studies [27, 31, 43] as well as non-experimental equine studies [45]. Furthermore, the observed differences could be a consequence of the qualitative comparison, which relied on reported findings. This could therefore be limited due to the detail of the analysis, reporting biases and/or interpretation. For example, although gliosis was a common finding, there was limited further characterisation of this in autopsies. [10, 20, 21, 24, 25, 36, 41, 47] Moreover, factors that could influence interpretation, such as the timing or duration of autopsy in relation to death, which are recognised to influence protein degradation and staining, were not reported. In contrast, animal studies explored gliosis in more detail, reporting more variable changes to glial cell types, such as oligodendrocytes and microglia [34, 52]. Pre-clinical experiments too are typically set up to investigate a specific hypothesis and features relevant for this systematic review may be under-reported. Comparison could also be limited by interpretation. For example, it was difficult to assess whether reported findings such as ‘myelin loss’ or ‘myelin pallor’ reflect primary demyelination or global axon loss, although this issue was shared with the benchmarked animal review [2].

However, these differences are noteworthy in the context of the known limitations of animal models and warrant further consideration. For example, most recent animal experiments have inserted prosthetics underneath the lamina, posterior to the spinal cord. This simulates solitary posterior compression which is an unusual feature of DCM in isolation [9]. Finite element analysis has typically shown maximal mechanical stress values in the surround of the compression site [23, 51]. In this context, that most prominent pathobiology reviews on DCM describe the onset of disease in the posterior horn [4, 8, 19], whereas human autopsies find the converse is potentially significant. Particularly given the prominence of motor dysfunction in clinical disease, a construct heavily weighted in the outcome measures of DCM [57].

Cavitation is more commonly reported after traumatic spinal cord injury [1, 6, 56]. This difference has been linked to the more significant destruction within the spinal cord from the high energy trauma but is recognised to evolve over time. The clinical status of patients identified in this study is difficult to ascertain, but the more prevalent finding of cavitation amongst DCM autopsies compared to pre-clinical models is unlikely to be explained by disease severity alone. A more likely explanation is that this reflects a chronicity of injury less easily simulated with animals,after all clinical studies report an average time to diagnosis and treatment from onset of symptoms of 2–5 years, but most DCM will currently go undiagnosed, and DCM in the short-term is rarely fatal [44].

Additionally, while necrosis was a commonly reported histological finding in the human studies included in this review, animal studies have overwhelmingly tended to report apoptosis. Whether this reflects experimental challenges of simulating chronic compression, real species differences or methodological differences in identifying the mechanism of cell loss is unclear. Abnormal autophagy, for example, has been linked to injury in DCM using human spinal cord specimen [16] but notably the only paper which used immunohistochemical techniques in this review (such as anti-Fas antibodies) specifically identified apoptosis [60]. This may suggest that the necrosis reported in older case series and case reports reflects experimental differences (i.e. identification via H&E and KB staining only rather than immunohistochemistry). Indeed, the inability to distinguish necrosis and apoptosis on standard histopathology sections means that dead cells tend to be categorised as ‘necrosis’ regardless of the pathway by which the cells died [33]. Certainly, combining the use of newer techniques which allow identification of specific histopathological processes with human tissue should provide invaluable insights.

Even if these nuances are true differences, they should not undermine the value of pre-clinical models. Animal studies offer obvious benefits of standardisation, experimental freedom, and large sample sizes. In contrast, as is evident in this systematic review, human autopsy studies are highly variable, less numerous and more inconsistent. They too, as aforementioned in the current context, are likely to reflect advanced disease. The ideal framework would therefore be a hybrid approach. This has greatly benefited other central nervous system diseases, particularly with the advent of more sophisticated molecular pathobiological techniques largely developed since most of the DCM autopsy studies have been conducted [12, 46, 18].

To this concept and potential, it is worth highlighting one study by Iwabuchi et al. (2004). [25] Published in the Fukushima Journal of Medical Science, it has received just 2 citations. However, the study reports on a detailed analysis of 68 autopsies, in which a histological diagnosis of DCM was made in 2 cases. This is interesting for several reasons. First, this offers a limited corroboration, using a different modality, to the epidemiology estimates by Smith et al. (2021) [49]. Due to widespread underdiagnosis, a true estimate of DCM prevalence has not been possible. Smith et al. (2021) aggregated healthy volunteer imaging studies and identified asymptomatic cervical cord compression in 24% of adults, and 2.3% prevalence of DCM. Iwabuchi et al. (2004) identified that 12 (18%) had evidence of cord compression, but only 2 (2.9%) histological features of DCM. However more importantly, it provides a histological series more analogous to clinical practice: not all cases with spinal cord compression acquired spinal cord injury, but this was more likely, and more severe with a higher compression ratio. This study therefore indicates the potential for human spinal cord specimen to compliment current research approaches in DCM.

### Limitations

A clear limitation of this systematic review was the inconsistent methodologies, diagnosis coding and reporting of the included studies. Although this is a well-known issue in systematic reviews, inconsistent coding and reporting styles are particular problems in the DCM field. The AO Spine RECODE-DCM aims to create a research toolkit to accelerate research development and improve patient outcomes through more consistent nomenclature and the setting of research priorities (aospine.org/recode) [15, 17, 39, 53].

Furthermore, due to the nature of included studies, no quantitative data was presented in any of the autopsy findings. This complicates an assessment of the importance of pathological findings, or how they co-exist. Few studies compared to controls, meaning it remains unclear which of the reported findings contribute to disease progression in DCM and which may be incidental in an ageing population. It is also notable that although a total of 150 autopsies were reported in the included studies, only 29% of these were on female patients, despite DCM affecting both men and women.

Due to the nature of the included studies being case reports or series of autopsies, consistent clinical data was lacking. In particular, as many patients were selected for inclusion after dying for unrelated reasons to DCM, most studies did not report individual data on duration of disease. Additionally, most papers were written and published before standard validated scoring systems for DCM symptom severity such as the modified Japanese Orthopaedic Association (mJOA) score were in use, limiting a clinical correlation to severity. Integration with other study types is therefore essential.

Finally, this article has focused on the potential role for human spinal cord tissue, and does not recognise complimentary insights that could arise from other tissue sources. For example, Laliberte et al. (2021) combined analysis of plasma miRNA in patients with DCM, with targeted experiments in animal and in vitro models to explore the significance of Mir21 expression in DCM outcomes. [32] Additionally, research into the use of biomarkers to monitor DCM progression is emerging, with raised CSF neurofilament light subunit (NF-L) and glial fibrillary acidic protein (GFAP) as well as lower amyloid β peptide being correlated with symptom duration [54]. Overall, this would align with our findings of the value to using a hybrid approach integrating different study types.

## Conclusion

Clearly, a knowledge gap exists in understanding the pathophysiology of DCM. While animal studies can offer experimental freedom and therefore key mechanistic insights, human autopsy studies offer the unique benefit of observing actual DCM histological changes. Integration and collaboration between pre-clinical and clinical research should therefore be a key priority towards understanding the pathophysiology of DCM and improving outcomes for patients.

## Supplementary information

Below is the link to the electronic supplementary material.

Supplementary file1 (DOCX 23 KB)

## Abbreviations

DCM: Degenerative cervical myelopathy
CSM: Cervical spondylotic myelopathy
H&E: Haematoxylin and eosin
OPLL: Ossification of the posterior longitudinal ligament
