# Supplementary material for: Human spinal cord tissue is an underutilised resource in degenerative cervical myelopathy: findings from a systematic review of human autopsies
Source: Acta Neurochir (Wien). 2023 Feb 23;165(5):1121–31. doi: 10.1007/s00701-023-05526-5 (PMC10140111; doi:10.1007/s00701-023-05526-5)
Supplement: Supplementary file 1 — Supplementary file1 (DOCX 23 KB) [file 701_2023_5526_MOESM1_ESM.docx]

**Supplementary Data 1 | PROSPERO registration**

**Review title**

Mechanistic insights into Degenerative Cervical Myelopathy from studies examining human spinal cord tissue: a systematic review

**Review question**

What are the findings of degenerative cervical myelopathy from studies evaluating human spinal cord specimen?

**Searches**

State the sources that will be searched (e.g. Medline). Give the search dates, and any restrictions (e.g. language or publication date). Do NOT enter the full search strategy (it may be provided as a link or attachment below.)

The search string will combine synonyms of Degenerative Cervical Myelopathy, human spinal cord and autopsy. It has been iterated with input from a medical librarian and will be applied to the following databases: Ovid MEDLINE and Ovid Embase. The search will be applied up to May 18, 2021.

**Condition or domain being studied**

Degenerative Cervical Myelopathy

**Participants/population**

Adult patients of any age with Degenerative Cervical Myelopathy

**Intervention(s), exposure(s)**

Any primary clinical study which reports findings from examination of human spinal cord specimen of deceased persons with degenerative cervical myelopathy.

**Types of study to be included**

Any peer-reviewed, published study which includes pathological findings of degenerative cervical myelopathy and its synonyms in human spinal cord will be included in our systematic review.

The review will exclude studies that: were not written in English; were not carried in human subjects; does not involve autopsy findings of human spinal cord; are systematic reviews, education papers, conference abstracts, protocols, reports and book chapters.

For clarity, the study focuses on human spinal cord tissue examined using miscroscopic / histological or other techniques. It does not include imaging studies of the live spinal cord for example.

**Main outcome(s)**

This systematic review will identify the existing findings of degenerative cervical myelopathy in human spinal cord on post-mortem examination.

**Measures of effect**

Will be determined based on the findings identified. Potential effect measures include the histological measures, such as the quantity of axonal loss or demyelination, as well as clinical disease measures such as disease severity scores (e.g. Japanese Orthopaedic Association or Nurick Score).

**Data extraction (selection and coding)**

Describe how studies will be selected for inclusion. State what data will be extracted or obtained. State how this will be done and recorded.

Studies will be selected in three stages. First, a pilot screening of 10% of the total studies yielded from the search strategy will be carried out by all reviewers, and reliability of study selection will be assessed on this sample. Subsequently, the remaining 90% of studies will be randomly divided among the reviewers such that each study has an exclusion/inclusion decision made by two reviewers. Following identification of potentially relevant studies, full-text screening will be carried out by all reviewers. In case of disagreement, an independent reviewer will have the deciding vote.

**Data extraction and strategy for data synthesis**

An extraction template will be developed by the investigators, and iterated following a pilot amongst included studies. Data extraction will be performed by two authors separately, with any disagreement settled via mutual discussion, or with input from a third investigator if required. A suitable risks of bias and quality assessment tool will be selected, once the nature of included studies is identified.

The analysis plan too will be informed by the data identified. It is likely that findings are heterogenous, and unsuitable for quantitative analysis. In this case a narrative synthesis, following the SWiM guidelines will be conducted. It is anticipated pathological findings could be grouped into key mechanistic themes, enabling a frequency comparison of their reported occurence.

**Supplementary Data 2 | Search strategy**

|  | **MEDLINE** |
| --- | --- |
| 1 | exp Cervical Vertebrae/ or exp Cervical Cord/ or cervical.tw |
| 2 | Exp Spinal Cord Diseases/ or Exp Spinal Diseases/ |
| 3 | degenerat*.tw |
| 4 | 2 and 3 |
| 5 | Myelopath*.tw |
| 6 | Myeloradiculopath*.tw |
| 7 | Radiculopath*.tw |
| 8 | Exp Spinal Cord Compression/ |
| 9 | Exp “Ossification of Posterior Longitudinal Ligament”/ |
| 10 | Ossification of the Posterior Longitudinal Ligament.tw |
| 11 | OPLL.tw |
| 12 | Exp Spinal Stenosis/ |
| 13 | Cervical stenosis.tw |
| 14 | Exp Spondylosis/ |
| 15 | Spondylosis.tw |
| 16 | Spondylotic.tw |
| 17 | Degenerative cervical myelopathy.tw |
| 18 | DCM.tw |
| 19 | Cervical spondylotic myelopathy.tw |
| 20 | CSM.tw |
| 21 | 4 or 5 or 6 or 7 or 8 or 9 or 10 or 11 or 12 or 13 or 14 or 15 or 16 or 17 or 18 or 19 or 20 |
| 22 | 1 and 21 |
| 23 | (Autops* or "postmortem exam*" or "post mortem exam*" or "post-mortem*").ti,ab. or exp *autopsy/ or exp *Cadaver/ or (cadaver* or corpse*).ti,ab. or (histopath* or histolog* or patholog*).ti,ab. or exp *pathology/ or exp *histology/ |
| 24 | 22 and 23 |
| 25 | animal/ not (human/ and animal/) |
| 26 | 24 not 25 |

| **EMBASE** | |  |
| --- | --- | --- |
| 1 | exp Cervical Vertebra/ or cervical spine/ or exp Cervical spinal cord/ or cervical.tw | |
| 2 | Exp *Spinal Cord Disease/ or Exp *Spine Disease/ | |
| 3 | Exp *degeneration/ | |
| 4 | degenerat*.tw | |
| 5 | 3 or 4 | |
| 6 | 2 and 5 | |
| 7 | Myelopath*.tw | |
| 8 | Myeloradiculopath*.tw | |
| 9 | Exp *radiculopathy/ | |
| 10 | Radiculopath*.tw | |
| 11 | Exp *Spinal Cord Compression/ | |
| 12 | Exp *Posterior Longitudinal Ligament/ and exp ossification/ | |
| 13 | Ossification of the Posterior Longitudinal Ligament.tw | |
| 14 | OPLL.tw | |
| 15 | Exp *vertebral canal stenosis/ | |
| 16 | Cervical stenosis.tw | |
| 17 | Exp *Cervical Spondylosis/ | |
| 18 | Exp *Spondylosis/ | |
| 19 | Spondylosis.tw | |
| 20 | Spondylotic.tw | |
| 21 | Exp *Cervical myelopathy/ | |
| 22 | Degenerative cervical myelopathy.tw | |
| 23 | DCM.tw | |
| 24 | Exp *Cervical spondylotic myelopathy/ | |
| 25 | Cervical spondylotic myelopathy.tw | |
| 26 | CSM.tw | |
| 27 | 6 or 7 or 8 or 9 or 10 or 11 or 12 or 13 or 14 or 15 or 16 or 17 or 18 or 19 or 20 or 21 or 22 or 23 or 24 or 25 or 26 | |
| 28 | 1 and 27 | |
| 29 | (Autops* or "postmortem exam*" or "post mortem exam*" or "post-mortem*").ti,ab. or exp *autopsy/ or exp *Cadaver/ or (cadaver* or corpse*).ti,ab. or (histopath* or histolog* or patholog*).ti,ab. or exp *pathology/ or exp *histology/ | |
| 30 | 29 and 28 | |
| 31 | animal/ not (human/ and animal/) | |
| 32 | 30 not 31 | |
